# Traditional rural values and posttraumatic stress among rural and urban undergraduates

**DOI:** 10.1371/journal.pone.0237578

**Published:** 2020-08-14

**Authors:** Emily M. Keller, Gina P. Owens

**Affiliations:** Department of Psychology, University of Tennessee Knoxville, Knoxville, Tennessee, United States of America; University of São Paulo, BRAZIL

## Abstract

Although rurality is often treated as an aspect of diversity, researchers disagree regarding whether the traditional rural values of self-reliance, distrust of outsiders, religiosity, centrality of family, and fatalism continue to differentiate rural versus urban undergraduates. The present study examined 1) whether differences in these values exist between rural and urban college students in the United States and 2) whether these rural values might mediate the association between geographic remoteness and posttraumatic stress symptom (PTSS) severity. College undergraduates in the United States who reported experiencing traumatic and/or stressful events (*N* = 213) completed measures of these constructs through an online survey. *T*-test results indicated that rural respondents had significantly higher levels of PTSS severity and distrust of outsiders and significantly lower levels of religiosity when compared with urban participants. After controlling for gender, distrust of outsiders and religiosity also emerged as significant mediators of the relationship between geographic remoteness and PTSS severity. Thus, despite research that highlights differences based on geographic location, similarities and differences exist for rural and urban undergraduates in the United States with regard to traditionally rural values. For rural undergraduate clients presenting with trauma symptoms, our results suggest that building trust and religious and/or spiritual self-care may be particularly critical.

## Introduction

Stressful events comprise major life events that impact psychological distress as well as traumatic incidents that meet the diagnostic criteria for posttraumatic stress disorder (PTSD) as defined by the Diagnostic and Statistical Manual of Mental Disorders 5^th^ edition (DSM-5) [1]. Thus, stressful events range from abandonment by family members [2, 3] to rape, sexual assault, and wartime violence [4]. Exposure to stress and trauma is relatively common in the United States. For example, research using a national sample in the United States (U.S.) has shown that approximately 90% of individuals have been exposed to trauma, while 8% meet the criteria for PTSD [5]. Epidemiological studies have indicated that women are at a higher risk of developing PTSD symptomology in comparison to men [6].

College undergraduates represent a population at risk of trauma exposure and associated symptoms. Research that specifically focused on college undergraduates found that over half (52%) of participants had been exposed to trauma and that roughly 7% were categorized as meeting full PTSD criteria [7]. In a series of studies of college students, prevalence rates of adverse life events ranged from approximately 56% to 85% [8]. Studies have indicated that a gender difference may also be present in college-aged populations such that female undergraduates experience more severe symptoms of PTSD following trauma exposure [9].

### Rural trauma

One contextual variable that may affect U.S. undergraduates who are exposed to stress and trauma is geographic location, specifically living in rural versus urban localities. The United States Census Bureau [10] identifies two types of urban areas: urbanized areas where at least 50,000 people reside and urban clusters containing at least 2,500 and less than 50,000 people. With this definition, roughly 19% of the U.S. population can be categorized as rural residents [11]. Researchers have recently begun to recognize rurality as an aspect of diversity characterized by its own behavioral norms and culture [12]. Despite this recent attention, research specifically investigating how aspects of rural culture may influence the experiences of college students with mental health needs has remained limited.

Some literature indicates that stressful or traumatic events may disproportionately affect mental health outcomes in rural college students. For example, using data from the National Crime Victimization Survey, Rennison, DeKeseredy, and Dragiewicz [13] found that U.S. rural women experienced intimate partner rape or sexual assault at rates that were three times higher than comparable urban women. Other research using U.S. national samples has found that rural and urban individuals experience at least similar rates of trauma [14]. Whealin et al [15] also found that a significantly higher number of rural veterans than urban veterans screened positively for a diagnosis of PTSD. Although a significant proportion of rural individuals experience trauma and may subsequently develop PTSD, few studies have investigated whether rural and urban undergraduates differ in terms of severity of posttraumatic stress symptoms (PTSS). This limited existing research has not utilized an undergraduate sample and has suggested that rather than rurality per se, certain values, such as religiosity, may better predict PTSS severity [16, 17]. Thus, a goal of the current study was to investigate whether PTSS severity differs based on rural status and whether potential rural values might better explain the potential relationship between rurality and PTSS severity.

### Rural values

Research consistently highlights the importance of rural culture in terms of mental healthcare and outcomes [18], but aside from self-reliance, rarely are rural values clearly identified and explicitly explored in terms of their contribution to mental health. Limited existing work has suggested that, in comparison to urban individuals, rural residents in the U.S. in general exhibit values such as “self-reliance, conservativism, a distrust of outsiders, religion, work orientation, emphasis on family, individualism, and fatalism” [19(pp37)]. Furthermore, media coverage and recent polls have highlighted a divided country regarding certain values based on geographic remoteness following the U.S. 2016 presidential election [20–22]. These articles have reported a renewal of self-sufficiency, independence, pride, and conservative values in rural areas. However, Wagenfeld et al [23] noted that disagreement appears in the literature about whether differences between rural and urban values exist, with some scholars arguing that dissimilarities may be decreasing. Thus, whether the potential rural values identified by Wagenfeld [19] may better represent older rather than younger generations of individuals living in rural areas in the U.S. is unclear [24]. In addition, we were unable to locate any quantitative studies comparing rural and urban values. Assessing potential differences is crucial since these cultural values could influence trauma outcomes. Therefore, another aim of this study was to examine whether values of self-reliance, distrust of outsiders, religion, family, and fatalism differentiate rural versus urban undergraduates. We also sought to identify whether these rural values may mediate the relationship between the geographic remoteness of one’s permanent residence and PTSS severity.

#### Self-reliance

One value that could distinguish rural and urban college students is self-reliance, defined at the rural community level as a preference for depending on oneself for help [25]. However, limited research exists examining the potential association between self-reliance and more negative outcomes after stress and trauma, such as PTSS severity. Although not directly assessing PTSS severity, Sousa [26] found that higher levels of self-reliance were associated with more negative effects of political violence on physical and mental health in a sample of adult Palestinian women from the West Bank. Among adolescents in the U.S., youths endorsing extremely high levels of self-reliance reported significantly higher symptoms of depression and suicidal ideation compared to individuals reporting lower levels of self-reliance [27]. No research was found examining levels of self-reliance and potential connections to PTSS severity in a rural undergraduate sample.

#### Distrust of others

Another variable that has received less attention in the literature and could impact trauma-related outcomes in U.S. rural populations is distrust/mistrust of others or cynicism. These terms are characterized by the overarching concept of hostility, wherein others are viewed as being motivated by selfishness and are considered to be a source of maltreatment [28]. Hostility has been broadly associated with negative psychological and physical health outcomes in research concerning correlates to coronary heart disease [29]. Furthermore, a meta-analysis found a large effect size between this more general concept of hostility and PTSD [30]. For distrust specifically, while we did not find any empirical research that focused on the association between these concepts and PTSS with rural residents or undergraduates, we were able to find one study that investigated the link between mistrust and PTSD among Vietnam veterans [31]. Results indicated that veterans who met PTSD criteria scored nearly one standard deviation higher on cynicism than veterans who did not meet PTSD criteria. As research is limited, the current study sought to clarify the relationship between distrust and PTSS severity based on geographic remoteness.

#### Religiosity

A third variable that could explain the potential relationship between the geographic region of origin and PTSS severity is religiosity. Religious or spiritual beliefs have been associated with PTSS severity in largely non-rural populations in the literature. Among undergraduates, negative religious coping, which is based on a belief in a condemning God and includes believing that traumatic events occur as a God-given punishment, was strongly related to higher rates of PTSD [32, 33]. Relatedly, another study found that religious beliefs that were associated with negative coping strategies were related to higher levels of PTSS severity in Iraq and Afghanistan veterans [34]. Using a combined sample of urban and rural residents, one study found that increased religiosity was associated with increased odds of being diagnosed with PTSD [16]. However, this relationship was non-significant for rural residents in isolation. Another study examined the relationships between religiosity and trauma with a rural community sample; however, this study focused on posttraumatic growth as an outcome rather than PTSS [35]. In this study, religious involvement served as a protective factor and was uniquely associated with an increase in the likelihood that participants would experience posttraumatic growth after a trauma. In light of these mixed findings, the current study sought to clarify the relationships between residency status, religiosity (defined as higher levels of religious involvement or activity) [36], and PTSS severity.

#### Emphasis on family

Emphasis on family may be another variable that differentiates rural and urban undergraduates in the U.S. Although we could find no research examining the relationship between traumatic events and how the centrality of family in rural areas is associated with posttraumatic adjustment, some related work seems to suggest that a relationship exists between mental health outcomes and familial support [37, 38]. Higher levels of family dysfunction have been associated with increased levels of PTSD symptomology among veterans [39]. For rural families specifically, Imig [38] found an inverse relationship between stress and family interactions, with higher stress associated with lower levels of family interaction. Although rural residents may emphasize family relationships [19], stress and trauma could possibly change those relationships such that familial closeness lessens following a traumatic experience [38].

#### Fatalism

Finally, research linking the rural value of fatalism, defined as a propensity to see destiny as defined by a higher power or dictated by a force other than free will [39], and trauma outcomes is limited, with limited research examining a potential association between fatalism and PTSS severity. One recent study found positive correlations between aspects of fatalism and PTSD symptom variables, such as re-experiencing and avoidance [39]. Other existing literature focuses on a specific type of stressor, namely, serious physical health issues. Gonzales et al [40] studied a sample of Latina immigrants with breast cancer and discovered that higher fatalism was associated with lower emotional well-being. Conversely, Keeley [41] conducted a qualitative study with a sample of low-income individuals from both rural and urban areas in the United States regarding their experiences with heart disease, lung cancer, diabetes, and depression. Interviewees connected fatalism with preventing stress by accepting rather than worrying about the future.

Due to limited research, clarification as to how self-reliance, a distrust of outsiders, religiosity, emphasis on family, and fatalism differ between U.S. rural versus urban undergraduates could be meaningful. Further, understanding whether these traditionally rural values mediate the association between geographic remoteness of residence and PTSS severity would be beneficial for clinicians treating rural young adults who present with trauma symptoms.

### The present study

Given the literature outlined above and the lack of research comparing rural and urban populations, the current study had multiple aims. First, we wanted to quantitatively assess for potential differences in PTSS severity and the historically rural values of self-reliance, distrust of outsiders, religion, emphasis on family, and fatalism in rural versus urban undergraduates in the U.S. Based on the literature, Hypothesis 1 was that participants who indicated that they were permanent residents of rural areas would score significantly higher on measures of PTSS severity and traditionally rural characteristics in comparison to individuals who permanently resided in urban areas. Second, to our knowledge, these potential rural values have never been examined as possible mediators that could help explain the association between geographic remoteness and PTSS severity. Accordingly, Hypothesis 2 was that rural characteristics would at least partially explain the relationship between geographic remoteness and PTSS severity. More specifically, geographic remoteness would be associated with higher levels of self-reliance, distrust, and fatalism, which would, in turn, be associated with higher levels of PTSS severity. Further, geographic remoteness would be associated with higher levels of religiosity and healthy family functioning, which would be associated with lower levels of PTSS severity.

## Methods

### Participants and procedure

Participants were 213 undergraduate students taking introductory psychology classes at a large Southeastern university in the United States who were recruited through the department of psychology research pool. Over half of participants were female (62%) with a mean age of 19.27 years (*SD* = 2.48). The majority of participants were Caucasian (79%), identified the Southeast as their geographic region of permanent residence (92%), and were first year students (62%). Participants were provided with the definition of rurality (a geographic area where less than 2,500 people reside) versus urbanicity (a geographic area composed of more than 2,500 residents) based on the classification system created by the United States Census Bureau.^10^ When participants self-identified the population density of their permanent residence based on these thresholds, approximately 26% of participants came from rural areas and 74% came from urban areas. Participants also identified their most traumatic event with the most common event being the sudden death of someone close to them (27%), followed by some other event that made them feel very scared, helpless, or horrified (16%); a severe transportation accident (9%); forced sexual contact as a child (9%); seeing someone die suddenly or get badly hurt or killed (9%); forced sexual contact as an adult (8%); sudden abandonment by a family member (8%); being hit or kicked hard enough to injure as a child (5%); sudden move (4%); and 2% or less each reported a natural disaster or fire, a bad accident at work or home, war trauma, being hit or kicked hard enough to injure as an adult, or attacked with a weapon. Additional demographic information divided by geographic region of origin can be found in Table 1. The average amount of time since the most traumatic event for participants was 3.99 years (*SD* = 4.04).

**Table 1:** Demographic information of study participants.

	Urban (*N* = 157)	Rural (*N* = 56)	Total (*N* = 213)
Gender			
Female	95	37	132
Male	62	19	81
Race			
Caucasian	122	46	168
African American	9	4	13
Asian American	8	0	8
Hispanic American/Latinx	10	2	12
Native American/First Nations/Native Alaskan	2	3	5
Multiracial/Other	6	1	7
Age			
18	70	18	88
19	53	21	74
20	19	9	28
21	8	2	10
22+	7	6	13
Year in School			
Freshmen	99	33	132
Sophomore	40	14	54
Junior	11	8	19
Senior	7	1	8

Potential participants read a brief description of the study requirements. To be eligible, participants had to be at least 18 years of age, have experienced a traumatic or stressful event, and claim permanent residency in the United States. After indicating interest in participating in the study and before beginning the survey, individuals viewed an informed consent document. Participants indicated their consent to participate by clicking yes to proceed to the online survey. All participants received one hour of experimental course credit, and all procedures were reviewed and approved by the University of Tennessee Institutional Review Board. The approval number was UTK IRB-17-03624-FB. Consent was obtained electronically, but data were analyzed anonymously, so names were not retained.

### Measures

#### PTSS severity

The PTSD Checklist-5 (PCL-5) [42] is a 20-item self-report measure reflecting DSM-5 [1] symptoms of PTSD. Items are rated according to how much a particular symptom has bothered the respondent over the past month from 0 (Not at all) to 4 (Extremely). Total scores range from 0 to 80, with higher scores indicating greater PTSD symptom severity. Example items include “repeated, disturbing, and unwanted memories of the stressful experience” and “blaming yourself or someone else for the stressful experience or what happened after it.” Weathers et al [42] recommends a cutoff of 33 or above for the total symptom severity score, suggesting probable PTSD. Prior research has found the PCL-5 to have high internal consistency in samples of war veterans and civilians with α = 0.95 for both [43]. Convergent validity with other measures of PTSD and divergent validity with scales not intended to measure PTSD also have been supported [44]. Internal consistency reliability in the current study was α = 0.95.

#### Trauma screening

The Trauma History Screen (THS) [3] is a 14-item measure that asks respondents to indicate whether they have experienced an event that could be considered traumatic by selecting “yes” or “no.” If they choose “yes,” participants indicate how many times they have experienced the event. Some example events include “a really bad car, boat, train, or airplane accident,” “attack with a gun, knife, or weapon,” and “seeing someone die suddenly or get badly hurt or killed.” As a minor modification of the THS, if more than one event was indicated by the participant, they also were asked to specify which event was most traumatic and how long ago this “most traumatic” event occurred. Undergraduates who indicated that they had not experienced any stressful or traumatic events according to the THS were not eligible to participate in the present study.

#### Self-reliance

The Self-Reliance Scale [45] measures the level of reluctance participants might have regarding pursuing assistance from others. This five-item subscale was developed as part of the Conformity to Masculine Norms Inventory-46, which is a shortened version of the Conformity to Masculine Norms Inventory [46]. Items are rated on a four-point Likert scale from 0 (Strongly disagree) to 3 (Strongly agree) with the total score ranging from 0 to 15. Higher scores indicate higher levels of self-reliance while lower scores signify less self-reliance. An example item is “It bothers me when I have to ask for help.” The self-reliance subscale has good internal reliability (α = 0.85) [45]. Evidence supports the convergent validity of the Self-Reliance Scale based on correlations with a similar measure of self-reliance (*r* = .24). Internal consistency reliability for the current study was α = 0.79.

#### Distrust of others

The 7-item Cynicism Scale [47] is designed to measure the degree to which individuals question messages imparted by management and believe that the companies for which they work will take advantage of them and is part of the Measures of Life Attitudes instrument. Kanter and Mirvis [47] define cynicism as having three components: the formulation of high expectations regarding self and others, feelings of disappointment towards oneself and other people, and a sense of betrayal and belief that one is being deceived by others. Although the scale has been used for organizational research purposes, participants are not primed for this setting in either the directions or items. Participants were asked to rate the extent to which they agree or disagree with statements related to how they may or may not feel about other people on a four-point Likert scale ranging from 1 (Strongly agree) to 4 (Strongly disagree). Scores range from 7 to 28 and were reverse scored such that higher scores indicated stronger distrust. Example items include, “Most people will tell a lie if they can gain by it” and “Most people are just out for themselves.” The scale has a moderately high degree of internal consistency (α = 0.78) [47] and moderate face validity [48]. Internal consistency reliability for this measure in the current study was α = 0.83.

#### Religiosity

The Duke University Religion Index (DUREL) [36] is a five-item instrument that measures religious involvement and contains three subscales: organizational religious activity, non-organizational religious activity, and intrinsic religiosity. Items are rated on a 5- to 6-point Likert-type scale with item-specific anchor points. Composite scores range from 5 to 27. Sample questions include “How often do you attend church or religious meetings?” and “How often do you spend time in private religious activities, such as prayer, meditation, or Bible study?” Psychometric investigations have supported combining all three subscales into a unidimensional factor scale [49], with higher scores indicating higher overall religiosity. The DUREL has high internal consistency (α = 0.91) and convergent validity of the subscales has been supported by correlations with similar measures of religiosity (*r =* -0.71 to -0.85) [49]. Internal consistency reliability was α = 0.91 in the current study.

#### Emphasis on family

The General Functioning Scale [50], which is designed to assess the overall health of the family, contains 12 items and is a subscale of the McMaster Family Assessment Device, a measure of family functioning. Items are rated on a four-point Likert scale, ranging from 1 (Strongly agree) to 4 (Strongly disagree). Items were reverse-scored such that higher scores designated healthy family functioning and lower scores signified unhealthy family functioning. Total scores range from 12 to 48. Example items include, “Individuals are accepted for who they are” and “We feel accepted for what we are.” The General Functioning Scale has adequate test-retest reliability *r* = 0.71 [51]. In addition, the General Functioning Scale exhibits good internal reliability (0.83 to 0.86) [52]. Byles et al [53] reported that the measure had good construct validity as it correlated with other family variables in their data pool. In the current study, internal consistency reliability was α = 0.91.

#### Fatalism

The Pearlin Mastery Scale [54] measures mastery or the extent to which people believe their lives are under their control rather than being fatalistically restrained. Items are rated on a four-point Likert scale ranging from 1 (Strongly disagree) to 4 (Strongly agree). Items were reverse-scored such that higher scores indicated lower levels of mastery and thus more fatalistic beliefs. Composite scores range from 7 to 28. Example items include, “I have little control over things that happen to me” and “Sometimes I feel that I am being pushed around in life.” Brady [55] indicated that the Pearlin Mastery Scale has strong face validity since it has been widely used and translated into other languages. The measure also has good internal consistency (α = 0.81) [56]. Internal consistency reliability for this measure in the current study was α = 0.78.

### Data analysis

Data analyses were conducted using SPSS software (version 25.0) [57]. To address Hypothesis 1, an independent samples *t*-test was performed to assess whether differences existed between undergraduate participants who claim permanent residency in rural versus urban areas regarding PTSS severity as well as the traditional rural values of self-reliance, distrust of outsiders, religiosity, centrality of family, and fatalism. In order to investigate the possible mediating role of historically rural values in the association between geographic remoteness and PTSS severity (Hypothesis 2), a mediation analysis was conducted using Hayes PROCESS [58] macro. To test significance, we implemented a bootstrapping analysis with 10,000 bootstrapping resamples to acquire 95% bias-corrected confidence intervals (CIs) for indirect effects [59, 60]. The indirect effects are determined to be significant if the CI does not contain zero [59].

## Results

Means, standard deviations, and internal consistency reliability were calculated for the variables of interest. Independent variables were checked for their appropriateness for multivariate analyses, and skewness, kurtosis, and multicollinearity were in acceptable ranges. Means, standard deviations, and intercorrelations among the variables of interest are presented in Table 2. Approximately 47% (46.9%) of participants scored at least 33 on the PCL-5, which is the cutoff for a probable diagnosis of PTSD [42]. According to bivariate correlational analyses, PTSS severity was significantly positively associated with geographic remoteness (*p* < .05). In addition, PTSS severity was significantly positively associated with self-reliance, distrust of outsiders, and fatalistic beliefs and significantly negatively associated with religiosity and emphasis on family (*p* < .01). Geographic remoteness was significantly positively associated with distrust of outsiders and significantly negatively associated with religiosity (*p* < .05). We examined gender as a potential covariate that might impact the outcome variable and found that gender (female = 0, male = 1) was significantly negatively correlated with PTSS severity (*p* < .01). Therefore, gender was entered as a covariate in the model.

**Table 2:** Means, standard deviations, and correlations among variables for total sample (N = 213).

Variables	M	SD	1	2	3	4	5	6	7
1. PTSS Severity	24.67	15.02	-	.15*	.22**	.31**	-.26**	-.33**	.41**
2. Geographic Remoteness				-	.11	.15*	-.14*	-.12	.10
3. Self-Reliance	11.57	2.71			-	.32**	-.13	-.30**	.34**
4. Cynicism	19.76	3.74				-	-.18**	-.14*	.27**
5. Religiosity	15.34	6.21					-	.24**	-.06
6. Family Emphasis	33.48	7.38						-	-.40**
7. Fatalism	15.69	3.60							-

PTSS Severity = PTSD Checklist-5; Geographic Remoteness (urban = 0, urban = 1); Family Emphasis = General Functioning Scale; Fatalism = The Pearlin Mastery Scale;

* *p* < .05,

** *p* < .01.

Significant differences were found for PTSS severity, with college students from rural areas reporting significantly higher PTSS severity than individuals from urban areas, *t*(211) = -2.27, *p* < .05, *d* = 0.35 (see Table 3). Regarding traditional rural values, the means were significantly different for distrust of outsiders and religiosity. Rural undergraduates indicated significantly higher levels of distrust of outsiders in comparison to urban students, *t*(211) = -2.16, *p* < .05. In addition, rural college students reported significantly lower levels of religiosity when compared to urban undergraduates, *t*(211) = 2.02, *p* < .05. No significant differences were found for the remaining values.

**Table 3:** Ranges, means, and standard deviations of undergraduates on PTSS severity and values.

Variables	Range	Rural		Urban		
		*M*	*SD*	*M*	*SD*	*t*
PTSS Severity	0–80	36.13	19.92	29.54	18.21	-2.27*
Self-Reliance	0–15	12.05	2.73	11.39	2.69	-1.57
Cynicism	7–28	20.68	3.89	19.43	3.64	-2.16*
Religiosity	5–27	13.91	6.67	15.85	5.98	2.02*
Family Emphasis	12–48	32.05	7.03	33.99	7.46	1.69
Fatalism	7–28	16.29	3.38	15.47	3.66	-1.46

PTSS Severity = PTSD Checklist-5; Family Emphasis = General Functioning Scale; Fatalism = The Pearlin Mastery Scale;

* = *p* < .05.

**Fig 1:**
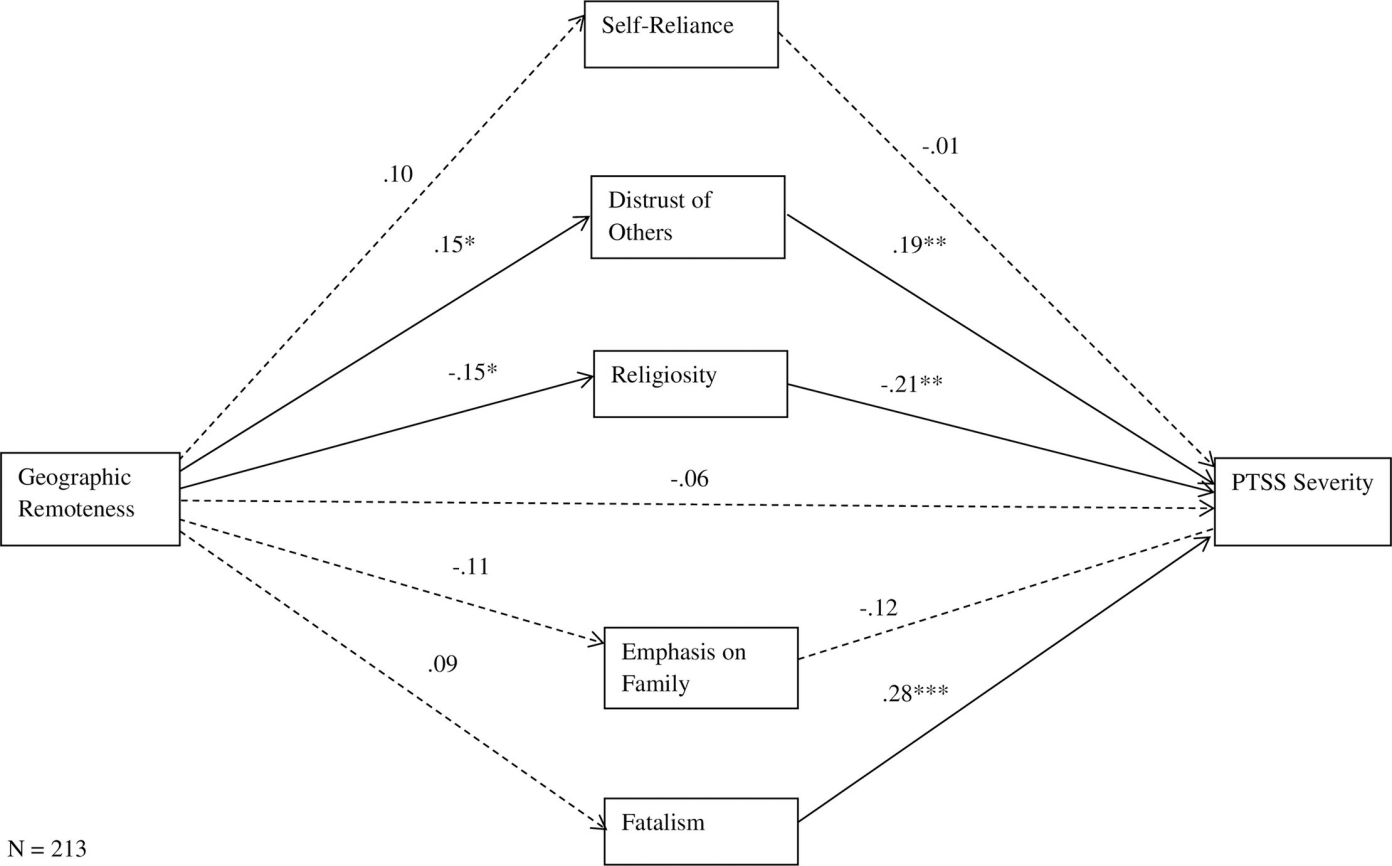
Path model of direct and indirect relations of variables of interest predicting PTSS severity. Gender was entered as a control variable but is not shown for ease of presentation. Values reflect standardized coefficients. *p < .05, **p < .01, *** *p* < .001.

For our mediation analysis, gender was entered as a covariate, geographic remoteness as the predictor, the rural values of self-reliance, distrust of others, religiosity, emphasis on family, and fatalism as mediators, and PTSS severity as the outcome variable. As shown in the mediation model (Fig 1), geographic remoteness (urban = 0, rural = 1) was significantly positively related to distrust of others (*p* < .05) and significantly negatively associated with religiosity (*p* < .05). PTSS severity was significantly positively correlated with distrust of others (*p* < .01) and fatalism (*p* < .001) and significantly negatively associated with religiosity (*p* < .05). Gender was significantly negatively correlated with religiosity (*B* = -.15), fatalism (*B* = -.15), and PTSS severity (*B* = -.18). Although rurality did not have a direct effect on PTSS severity, distrust of others (mean indirect effect [unstandardized] = 1.17, SE = .75, 95% CI [.01, 2.91], β = .03) and religiosity (mean indirect effect [unstandardized] = 1.24, SE = .73, 95% CI [.03, 2.87], β = .03) arose as significant indirect effects between geographic remoteness and PTSS severity. No other indirect effects were found for remaining historically rural characteristics. The variables explained 30% of the variance in PTSS severity.

## Discussion

Based on largely qualitative prior research, the present study sought to explore potential differences attributed to geographic remoteness of permanent residence on PTSS severity and the historically rural values of self-reliance, distrust of outsiders, religiosity, centrality of family, and fatalism using quantitative measures in a sample of undergraduates. In addition, the present study investigated whether these traditionally rural values mediated the relationship between geographic remoteness and PTSS severity. In partial support of our first hypothesis, *t*-test results indicated significant differences in terms of PTSS severity, distrust of outsiders, and religious activity. No significant differences based on rural versus urban status were found for self-reliance, emphasis on family, and fatalism. Regarding our second hypothesis, as predicted, distrust of outsiders and religiosity mediated the relationship between geographic remoteness and PTSS severity when controlling for gender. However, contrary to our hypothesis, none of the other historically rural values were significant mediators. Therefore, our second hypothesis was also partially supported.

With regard to our first hypothesis, our findings suggest that U.S. rural undergraduates display higher PTSS severity in comparison to urban students. In our sample, the number of traumatic events reported by rural (*M* = 8.72, *SD* = 14.86) versus urban (*M* = 6.47, *SD* = 12.35) undergraduates was not significantly different, providing further support for this finding. The current study did not explore treatment options; therefore, one potential implication for this difference could be that trauma-focused treatments are challenging to implement due to certain geographical and financial obstacles in rural areas that impact both clients and practitioners. According to Mohatt et al [18], rural persons with mental illnesses experience difficulties with accessing healthcare. The innate isolation of rural areas complicates travel options, and many rural individuals experience difficulties with affording health services. A shortage of rural mental health providers also exists, which impacts the availability of services even when clients have the desire to access healthcare. The limited anonymity that comes with living in an area with low population density may also significantly lessen the chance that rural residents will seek psychological services [18]. With these obstacles to seeking help, rural individuals who experience PTSS following a trauma may not be able to receive support in comparison to individuals in urban areas.

Most rural participants in the current study were presently living in an urban environment to attend college. Since our sample largely consisted of freshmen, it is possible that a lack of availability of healthcare resources at home might have contributed to current mental health outcomes. This prior lack of resources could have hindered their recovery despite currently living in an urban environment where campus mental healthcare resources were more readily available. Furthermore, the stigma associated with seeking help, which may be especially prevalent in rural localities [18], may have kept rural undergraduates from pursuing campus healthcare resources. While these questions are beyond the scope of the current study, future research should investigate the potential impact of mental healthcare availability and acceptability prior to college attendance on mental health outcomes in rural undergraduates.

Aside from PTSS severity, rural undergraduates reported higher levels of distrust for outsiders and lower levels of religiosity in comparison to urban participants, providing partial support for Hypothesis 1. Our results related to distrust of outsiders confirmed prior research [19, 23] that suggested this characteristic has historically differentiated rural from urban residents. However, an unexpected result concerned the significantly higher religiosity scores of urban participants compared to rural participants. Perhaps urban undergraduates were accustomed to finding a church in an urban environment while rural college students were less familiar with locating an organized religious institution in a city setting. Another possible explanation is that the county of the permanent residence of approximately 19% of participants matched the county of their university, meaning that many of these urban undergraduates would potentially not have had to locate a new religious institution upon transitioning to a college environment, unlike permanent residents of rural areas who chose to attend church. In addition, rural individuals may be resistant to churches in cities, which may be larger or emphasize different practices or traditions than the churches to which they are accustomed [61].

The undergraduate status of the participants may also have affected the results. In general, rural Southerners in the United States, who made up the primary location demographic in our study, tend to display more religiosity and conservatism in comparison to rural communities in other areas of the United States [62]. Furthermore, approximately 85% of participants permanently resided in one of the top three states that the Pew Research Center [63] has characterized as the mostly highly religious states in the United States. The Pew Research Center [64] has also found that, while 72% of college undergraduates categorize religion as very or somewhat important, only 36% of college students attend church at least once per week. As our measure of religiosity included a question about organizational religious activity, which rural participants may already be less inclined to attend in an urban environment, our results may have been affected. Therefore, our finding that rural Southeastern undergraduates display less religiosity than their urban counterparts may be unique to students attending urban-based universities in the Southeastern United States.

Aside from distrust of outsiders and religious activity, our findings indicate that the historical differences in values for rural versus urban undergraduates might be dwindling. For the last several decades, some academic researchers have theorized that the divide in values for U.S. rural and urban residents may be lessening [19], although research concerning the values of interest in the present study tends to be dated and published before the 2000s [23]. While the present study supports the idea that distrust of outsiders continues to differentiate rural areas from urban areas, these differences were not present for any of the other variables, except for religiosity, which showed differences that were opposite of the predicted direction. However, although few significant differences regarding traditionally rural values were found for our undergraduate sample, these values do characterize both rural and urban young adults. Religion and fatalism also appeared to be important to students who were permanent residents of both geographic residences. Thus, our results suggest that while geographic remoteness may not be as dichotomizing as some prior research has indicated, these cultural values do continue to be emphasized in rural locations. However, as our sample was limited to undergraduates who permanently resided in the southeastern United States, future research should target rural community members and other rural locations, such as the Midwest.

One explanation for the discovery of more similarities in this study can be found in what Lichter and Brown [65] call the accelerating urbanization of rural society whereby the boundaries between rural and urban society are increasingly blurring. American rural life has traditionally been associated with agriculture, but between 1940 and 1992, the population of farmers decreased from 30 million to 3.9 million people [66]. With this agricultural deterioration, communities that remained agriculturally dependent experienced extensive population decline as former farmers migrated from these areas. Additionally, non-farming businesses were forced to close as their farming clientele moved away [66]. Transnational corporations, such as Wal-Mart, started to invest and appear in rural communities in the 1960s, further contributing to the decline of small businesses and the downtown areas of rural towns [67, 68]. Further, innovations such as the Internet, cable and satellite TV, and broadband have promoted the rapid transformation of information, linking rural to urban life [65].

In addition to these general changes that may impact all generations of rural populations, it is important to consider that rural areas consist of subpopulations that may not be acculturated with characteristically rural customs, traditions, and values [69]. Young adults, who composed the majority of our participant population, may have unique characteristics in rural areas in comparison to older members of these communities [62, 70]. When individuals have migrated from rural areas to urban areas, rural young adults appear to be particularly attracted to moving to urban residences, perhaps due to social and economic advantages, and thus display the highest incidence of migration [69].

Differences regarding adherence to or internalization of rural values could also be impacted by the diversity of rural areas [18]. Our rural sample primarily represented Appalachia, but rural areas can also be found in frontier America located in the Western United States [15] and in small towns off interstates [18]. In the Eastern part of the United States, rural America is more densely populated, while in the Western part of the United States, rural areas are more remote to the extent that availability of healthcare is even more challenging [18, 19]. It could also be that the historical rural values of interest characterize Appalachian culture in general [71] rather than broadly defined rural culture. Appalachian culture has traditionally counted independence, mistrust of others, family ties, and religiosity as integral parts of Appalachian identity [71].

Our second hypothesis was partially supported with two rural values, distrust of outsiders and religiosity, mediating the relationship between geographic remoteness and PTSS severity when controlling for gender. Limited existing research often supported conflicting conclusions linking many of these variables. Due to the importance of distrust, mental healthcare professionals and campus healthcare providers who work with rural residents identified as having symptoms of PTSD should devote time to building rapport and discussing potential misconceptions that rural clients may have about trust of others both in the therapy room and with people in their everyday lives. Interventions that focus on building trust and relationships with others may be particularly critical for individuals who have experienced stressful events and/or symptoms of PTSD. It may be especially important for student counseling centers and other campus medical providers to participate in outreach events. Outreach events and other forms of on-campus advocacy efforts that bring attention to mental healthcare resources could lessen distrust and suspicion of mental healthcare professionals on and off campus and promote awareness of healthcare resources that might not be available near the permanent residences of rural students.

In addition, higher levels of religiosity may play a protective role when undergraduates experience traumatic or stressful events. Positive religious coping techniques, including praying for relief, seeking comfort in a faith community, and believing that God will provide divine justice in the wake of traumatic events [32]. may be particularly helpful. Mental healthcare professionals and campus healthcare providers might consider encouraging students, especially rural college students, to connect with their college communities by attending worship services. Other methods of self-care that relate to religion or spirituality, such as prayer or mediation, may also provide a protective buffer for PTS symptoms.

### Limitations and future directions for research

The current study is subject to certain limitations that should be taken into consideration. As already indicated, our sample comprised undergraduates who were taking classes at a university located in an urban environment, so it is unclear whether these results would extend to rural college students taking courses in a rural area. Although participants were required to have experienced a traumatic event, some of these individuals may have experienced stressful events that did not meet Criterion A of the DSM-5 for PTSD [1], which could have affected PTSS severity. The mean time since the traumatic event had occurred was 3.99 years, which may have affected PTSS severity. In addition, the exclusion of participants who had not experienced stressful or traumatic events could have contributed to the finding of fewer differences between rural and urban undergraduates regarding traditional rural values than was predicted.

Future research would benefit from more diversity in terms of ethnicity, race, and region of residence. In addition, future research should also include other rural regions of interest, such as frontier America, within their sample. While the proportion of rural undergraduates in the study (26%) was comparable to the total rural population in the United States (19%) [10], future research should attempt to recruit a larger percentage of rural participants. Further, in the current study, rural undergraduates self-identified geographic remoteness based on the United States Census Bureau definition [10], meaning that rural status was subjective.

Another limitation of the present study involved the measures that were used. It was difficult to locate measures for distrust of outsiders, centrality of family, and fatalism, in particular. In addition, the Cynicism Scale [47] was designed for evaluating workforce samples, but the questions were phrased so that they could apply to other populations. Some of the questions from the General Functioning Scale [50] concerned emphasis on family, but the scale was designed to determine family functioning.

## Conclusion

Despite these limitations, our findings underscore notable differences and similarities regarding PTSS severity and the historically rural values of self-reliance, distrust of outsiders, religiosity, centrality of family, and fatalism based on geographic remoteness. Rural undergraduates in the U.S. exhibited higher levels of PTSS severity and distrust of outsiders, while urban students displayed a higher degree of religiosity. However, there were no significant differences for the other traditionally rural values. In addition, when controlling for gender, distrust of others and religiosity emerged as significant mediators of the association between geographic remoteness and PTSS severity. The results suggest relevant constructs and interventions that clinicians should recognize when considering PTSS severity in rural young adults. In addition, the findings highlight the importance of further exploring specific cultural factors that differentiate rural and urban undergraduates and that could affect mental health outcomes in U.S. college populations.

## References

[1] American Psychiatric Association. Diagnostic and statistical manual of mental disorders. 5^th^ ed Washington, D. C.: Author 2013.

[2] Anders SL, Shallcross SL, Frazier PA. Beyond criterion A1: the effects of relational and non-relational traumatic events. J Trauma Dissociation. 2012;13(2):134–151. doi: 10.1080/15299732.2012.642744 22375804

[3] Carlson EB, Smith SR, Palmieri PA, Dalenberg C, Ruzek JI, Kimerling R, et al Development and validation of a brief self-report measure of trauma exposure: the trauma history screen. Psychol Assess. 2011; 23(2):463–477. doi: 10.1037/a0022294 21517189 PMC3115408

[4] Briere J, & Scott C. Principles of trauma therapy: a guide to symptoms, evaluation, and treatment. New York: SAGE Publications 2006.

[5] Kilpatrick DG, Resnick HS, Milanak ME, Miller MW, Keyes KM, Friedman MJ. (2013). National estimates of exposure to traumatic events and PTSD prevalence using DSM-IV and DSM-5 criteria: DSM-5 PTSD prevalence. J Trauma Stress. 2013;26(5):537–547. doi: 10.1002/jts.21848 24151000 PMC4096796

[6] Olff M, Langeland W, Draijer N, Gersons BPR. Gender differences in posttraumatic stress disorder. Psychol Bull. 2007;133(2):183–204. doi: 10.1037/0033-2909.133.2.183 17338596

[7] Bergman HE, Kline AC, Feeny NC, Zoellner LA. Examining PTSD treatment choice among individuals with subthreshold PTSD. Behav Res Ther. 2015;73:33–41. doi: 10.1016/j.brat.2015.07.010 26246029 PMC4573338

[8] Smyth JM, Hockemeyer JR, Heron KE, Wonderlich SA, Pennebaker JW. (2008). Prevalence, type, disclosure, and severity of adverse life events in college students. J Am Coll Health. 2008;57(1):69–76. doi: 10.3200/JACH.57.1.69-76 18682348

[9] Moser JS, Hajcak G, Simons RF, Foa EB. Posttraumatic stress disorder symptoms in trauma-exposed college students: the role of trauma-related cognitions, gender, and negative affect. J Anxiety Disord. 2007;21:1039–1049. doi: 10.1016/j.janxdis.2006.10.009 17270389 PMC2169512

[10] United States Census Bureau. *Urban and rural* Available from: https://www.census.gov/programs-surveys/geography/guidance/geo-areas/urban-rural.html [Accessed 28 December 2019].

[11] Ratcliffe M, Burd C, Holder K, Fields A. *Defining rural at the US Census Bureau: American Community Survey and Geography Brief (ACSGEO-1)* Available from: http://www.census.gov/content/dam/Census/library/publications/2016/acs/acsgeo-1.pdf [Accessed 28 December 2019].

[12] Smalley KB, Warren JC. Rurality as a diversity issue In: Smalley KB, Rainer JP, editors. Rural mental health: issues, policies, and best practices. New York, NY, US: Springer Publishing Company; 2012 p. 37–47.

[13] Rennison CM, DeKeseredy WS, Dragiewicz M. Urban, suburban, and rural variations in separation/divorce rape/sexual assault: results from the National Crime Victimization Survey. Fem Criminol. 2012;7(4):282–297. doi: 10.1177/1557085111435660

[14] McCall-Hosenfeld JS, Mukherjee S, Lehman EB. The prevalence and correlates of lifetime psychiatric disorders and trauma exposures in urban and rural settings: Results from the National Comorbidity Survey Replication (NCS-R). PLoS ONE, 2014;9(11):1–11. doi: 10.1371/journal.pone.0112416 25380277 PMC4224442

[15] Whealin JM, Stotzer RL, Pietrzak RH, Vogt D, Shore J, Morland L, et al Deployment-related sequelae and treatment utilization in rural and urban war veterans in Hawaii. Psychol Serv. 2014;11:114–123. doi: 10.1037/a0032782 24099457

[16] Erickson LD, Hedges DW, Call VRA, Bair B. Prevalence of and factors associated with subclinical posttraumatic stress symptoms and PTSD in urban and rural areas of Montana: a cross-sectional study. J Rural Health. 2013;29:403–412. doi: 10.1111/jrh.12017 24088214

[17] Thorne K, Ebener D. Locus of control as a mediator between posttraumatic stress and suicide risk: rural implications. Rural Soc. 2018; 27(3):208–223. doi: 10.1080/10371656.2018.1504759

[18] Mohatt DF, Adams SJ, Bradley MM, Morris CD, editors. Mental health and rural America, 1994–2005: an overview and annotated bibliography. 3rd edition Rockville, MD: Health Resources and Services Administration, Office of Rural Health Policy; 2006.

[19] Wagenfeld MO. A snapshot of rural and frontier America In: Stamm BH, editor. Rural behavioral health care: an interdisciplinary guide. Washington, D.C.: American Psychological Association; 2003 p. 33–40.

[20] DelReal JA, Clement S. Rural divide. The Washington Post. 2017 Jun 17. Available from: https://www.washingtonpost.com/

[21] Hamel L, Wu B, Brodie M. The health care views and experiences of rural Americans: findings from the Kaiser Family Foundation/Washington Post survey of rural America. 2017, Jun 16. Available from: https://www.kff.org/health-reform/report/the-health-care-views-and-experiences-of-rural-americans-findings-from-the-kaiser-family-foundationwashington-post-survey-of-rural-america/

[22] Richards K. What advertisers need to know about values in rural America today. Adweek. 2017, Feb 9. Available from: https://www.adweek.com/agencies/what-advertisers-need-to-know-about-values-in-rural-america-today/

[23] Wagenfeld MO, Murray JD, Mohatt D, DeBruyn JC. Mental health and rural America: 1980–1993. Washington, DC: U.S. Government Printing Office; 1994.

[24] Dorfman LT, Murty SA, Evans RJ, Ingram JG, Power JR. History and identity in the narratives of rural elders. J Aging Stud. 2004;18(2):187–203. doi: 10.1016/j.jaging.2004.01.004

[25] Green GD. Training for self-reliance in rural areas. Int Labour Rev. 1981; 120(4)411–423.

[26] Sousa CA. Political violence, health, and coping among Palestinian women in the West Bank. Am J Orthopsychiatry. 2013;83(4):505–519. doi: 10.1111/ajop.12048 24164522 PMC4545659

[27] Labouliere C, Kleinman M, Gould M. When self-reliance is not safe: associations between reduced help-seeking and subsequent mental health symptoms in suicidal adolescents. Int J Environ Res Public Health. 2015;12(4):3741–3755. doi: 10.3390/ijerph120403741 25837350 PMC4410213

[28] Smith TW. Concepts and methods in the study of anger, hostility, and health In: Siegman AW, Smith TW, editors. Anger, hostility, and the heart. Hillsdale, NJ, US: Lawrence Erlbaum Associates, Publishers; 1994 p. 23–42.

[29] Smith TW, Glazer K, Ruiz JM, Gallo LC. Hostility, anger, aggressiveness, and coronary heart disease: an interpersonal perspective on personality, emotion, and health. J Pers. 2004;72(6):1217–1270. 10.1111/j.1467-6494.2004.00296.x 15509282

[30] Orth U, Wieland E. Anger, hostility, and posttraumatic stress disorder in trauma-exposed adults: a meta-analysis. J Consult Clin Psychol. 2006; 74(4):698–706. 10.1037/0022-006X.74.4.698 16881777

[31] Kubany ES, Gino A, Denny NR, Torigoe RY. Relationship of cynical hostility and PTSD among Vietnam veterans. J Trauma Stress. 1994;7(1):21–31. Available from: https://onlinelibrary.wiley.com/journal/15736598 10.1007/BF02111909 8044439

[32] Bryant-Davis T, Wong EC. Faith to move mountains: religious coping, spirituality, and interpersonal trauma recovery. Am Psychol. 2013;68(8):675–684. doi: 10.1037/a0034380 24320650

[33] Gerber MM, Boals A., Schuettler D. The unique contributions of positive and negative religious coping to posttraumatic growth and PTSD. Psychol Relig Spirit. 2011;3(4):298–307. doi: 10.1037/a0023016

[34] Park CL, Smith PH, Lee SY, Mazure CM, McKee SA, Hoff R. Positive and negative religious/spiritual coping and combat exposure as predictors of posttraumatic stress and perceived growth in Iraq and Afghanistan veterans. Psycholog Relig Spiritual. 2017;9(1):13–20. doi: 10.1037/rel0000086 28217246 PMC5310632

[35] Hamby S, Grych J, Banyard V. Resilience portfolios and poly-strengths: identifying protective factors associated with thriving after adversity. Psychol Violence. 2018;8(2):172–183. doi: 10.1037/vio0000135

[36] Koenig HG, and Büssing A. The Duke University Religion Index (DUREL): A five-item measure for use in epidemological studies. Religions. 2010;1(1):78–85. doi: 10.3390/rel1010078

[37] Evans L, Cowlishaw S, Hopwood M. Family functioning predicts outcomes for veterans in treatment for chronic posttraumatic stress disorder. J Fam Psychol. 2009;23(4):531–539. doi: 10.1037/a0015877 19685988

[38] Imig DR. Urban and rural families: a comparative study of the impact of stress on family interaction. Rural Educ. 1983;1(2):43–46.

[39] Maercker A, Ben-Ezra M, Esparza OA, Augsburger M. Fatalism as a traditional cultural belief potentially relevant to trauma sequelae: measurement equivalence, extent and associations in six countries. Eur J Psychotraumatol. 2019;10(1). doi: 10.1080/20008198.2019.1657371 31528270 PMC6735334

[40] Gonzales FA, Hurtado-de-Mendoza A, Santoyo-Olsson J, Nápoles AM. Do coping strategies mediate the effects of emotional support on emotional well-being among Spanish-speaking Latina breast cancer survivors? Psychooncology. 2016;25(11):1286–1292. doi: 10.1002/pon.3953 26352186 PMC4785099

[41] Keeley B, Wright L, Condit CM. Functions of health fatalism: fatalistic talk as face saving, uncertainty management, stress relief and sense making. Sociol Health Illn. 2009;31(5):734–747. doi: 10.1111/j.1467-9566.2009.01164.x 19392939

[42] Weathers FW, Litz BT, Keane TM, Palmieri PA, Marx BP, Schnurr PP. The PTSD Checklist for DSM–5 (PCL-5). Available from: http://www.ptsd.va.gov/professional/assessment/adult-sr/ptsd-checklist.asp [Accessed 28 December 2019].

[43] Armour C, Tsai J, Durham TA, Charak R, Biehn TL, Elhai JD, et al Dimensional structure of DSM-5 posttraumatic stress symptoms: support for a hybrid Anhedonia and Externalizing Behaviors Model. J. Psychiatr Res. 2015;61:106–113. doi: 10.1016/j.jpsychires.2014.10.012 25479765

[44] Wortmann JH, Jordan AH, Weathers FW, Resick PA, Dondanville KA, Hall-Clark B, et al Psychometric analysis of the PTSD Checklist-5 (PCL-5) among treatment-seeking military service members. Psychological Assessment. 2016;28(11):1392–1403. doi: 10.1037/pas0000260 26751087

[45] Parent MC, Moradi B. Confirmatory factor analysis of the Conformity to Masculine Norms Inventory and development of the Conformity to Masculine Norms Inventory-46. Psychol Men Masc. 2009;10(3):175–189. doi: 10.1037/a0015481

[46] Mahalik JR, Locke B, Ludlow L, Diemer M, Scott RPJ, Gottfried M, et al Development of the Conformity to Masculine Norms Inventory. Psychol Men Masc. 2003;4(1):3–25. doi: 10.1037/1524-9220.4.1.3

[47] Kanter DL, Mirvis PH. The cynical Americans: living and working in an age of discontent and disillusion. San Francisco: Jossey-Bass; 1989.

[48] Stanley DJ. Employee cynicism about organizational change: development and validation of a measure. University of Western Ontario. Available from: http://www.collectionscanada.gc.ca/obj/s4/f2/dsk3/ftp04/mq30850.pdf [Accessed 28 December 2019].

[49] Storch EA, Roberti JW, Heidgerken AD, Storch JB, Lewin AB, Killiany EM, et al The Duke Religion Index: a psychometric investigation. Pastoral Psychol. 2004;53(2):175–181. Available from: https://link.springer.com/journal/11089

[50] Epstein NB, Baldwin LM, Bishop DS. The McMaster Family Assessment Device. J Marital Fam Ther. 1983;9(2):171–180. doi: 10.1111/j.1752-0606.1983.tb01497.x

[51] Miller IW, Epstein NB, Bishop DS, Keitner GI. The McMaster Family Assessment device: reliability and validity. J Marital Fam Ther. 1985;11(4):345–356. Available from: http://onlinelibrary.wiley.com/journal/10.1111/(ISSN)1752-0606

[52] Kabacoff RI, Miller IW, Bishop DS, Epstein NB, Keitner GI. A psychometric study of the McMaster Family Assessment Device in psychiatric, medical, and nonclinical samples. J Fam Psychol. 1990;3(4):431–439. http://www.apa.org/pubs/journals/fam/

[53] Byles J, Byrne C, Boyle MH Offord DR. (1988). Ontario Child Health Study: reliability and validity of the general functioning subscale of the McMaster Family Assessment Device. Fam Process. 1988;27(1):97–104. Available from: http://onlinelibrary.wiley.com/journal/10.1111/(ISSN)1545-5300 10.1111/j.1545-5300.1988.00097.x 3360100

[54] Pearlin LI, Schooler C. The structure of coping. J Health Soc Behav. 1978;19(1):2–21. doi: 10.2307/2136319 649936

[55] Brady TJ. Measures of self-efficacy, helplessness, mastery, and control: the Arthritis Helplessness Index (AHI)/Rheumatology Attitudes Index (RAI), Arthritis Self-Efficacy Scale (ASES), Children’s Arthritis Self-Efficacy Scale (CASE), Generalized Self-Efficacy Scale (GSES), Mastery Scale, Multi-Dimensional Health Locus of Control Scale (MHLC), Parent’s Arthritis Self-Efficacy Scale (PASE), Rheumatoid Arthritis Self-Efficacy Scale (RASE), and Self-Efficacy Scale (SES). Arthritis Rheum. 2003;49(5);S147–S164. doi: 10.1002/art.11413

[56] van Zoonen K, Kleiboer A, Cuijpers P, Smit J, Penninx B, Verhaak P, et al Determinants of attitudes towards professional mental health care, informal help and self-reliance in people with subclinical depression. Int J Soc Psychiatr. 2016;62(1):84–93. Available from: http://journals.sagepub.com/home/isp 10.1177/0020764015597014 26243151

[57] IBM Corp. IBM SPSS Statistics for Windows, Version 25.0 [software]. Armonk, NY: IBM Corp; 2017.

[58] Hayes AF. Introduction to mediation, moderation, and conditional process analysis: a regression-based approach. 2nd ed New York: Guilford Press; 2017.

[59] Preacher KJ, Hayes AF. Asymptotic and resampling strategies for assessing and comparing indirect effects in multiple mediator models. Behav Res Methods, 2008;40(3):879–891. doi: 10.3758/brm.40.3.879 18697684

[60] Shrout PE, Bolger N. Mediation in experimental and nonexperimental studies: new procedures and recommendations. Psychol Methods. 2002;7(4):422–445. doi: 10.1037//1082-989X.7.4.422 12530702

[61] Ross B Jr. Is there a rural-urban divide in the church? The Christian Chronicle. 2007 4 1 Available from: https://christianchronicle.org/is-there-a-rural-urban-divide-in-the-church/

[62] Dillon M, Savage S. *Values and religion in rural America*: *attitudes toward abortion and same-sex relations (Issue Brief No*. *1)*. Durham, NH: The Carsey Institute at the University of New Hampshire; 2006 Available from: https://scholars.unh.edu/carsey/12 [Accessed 28 December 2019].

[63] Pew Research Center. How religious is your state? 2016, Feb 29. Available from: https://www.pewresearch.org/fact-tank/2016/02/29/how-religious-is-your-state/?state=alabama

[64] Pew Research Center. Religious landscape study: college graduates. 2015. Available from: https://www.pewforum.org/religious-landscape-study/educational-distribution/college/

[65] Lichter DT, Brown DL. Rural America in an urban society: Changing spatial and social boundaries. Annu Rev Sociol. 2011;37:565–592. doi: 10.1146/annurev-soc-081309-150208

[66] Albrecht DE. The industrial transformation of farm communities: Implications for family structure and socioeconomic conditions. Rural Sociol. 1998;63(1):51–64. doi: 10.1111/j.1549-0831.1998.tb00664.x

[67] Stone KE. Impact of the Wal-Mart phenomenon on rural communities. Chicago, IL: Farm Foundation; 1997.

[68] Vias AC. Bigger stores, more stores, or no stores: paths of retail restructuring in rural America. J Rural Stud. 2004;20(3):303–318. doi: 10.1016/j.jrurstud.2003.10.003

[69] Slama K. Rural culture is a diversity issue. Minnesota Psychol. 2004;53:9–13. Available from: https://apa.org/practice/programs/rural/rural-culture.pdf

[70] Johnson KM. *Demographic trends in rural and small town America (Vol*. *1*, *No*. *1)*. Durham, NH: The Carsey Institute at the University of New Hampshire; 2006 Available from: https://scholars.unh.edu/cgi/viewcontent.cgi?article=1004&context=carsey [Accessed 28 December].

[71] Tang M, Russ K. Understanding and facilitating career development of people of Appalachian culture: An integrated approach. Career Dev Q. 2007;56(1):34–46. doi: 10.1002/j.2161-0045.2007.tb00018.x

